# Exercise-Induced Colon Ischemia in a Middle-Aged Female Athlete: Response to a Novel Therapeutic Approach of Sildenafil and Fludrocortisone

**DOI:** 10.14309/crj.0000000000001864

**Published:** 2025-10-20

**Authors:** Sanjay R.V. Gadi, Lawrence J. Brandt, Jatin Roper

**Affiliations:** 1 Department of Medicine, Duke University School of Medicine, Durham, NC; 2 Department of Medicine, Division of Gastroenterology, Albert Einstein College of Medicine, Bronx, NY; 3 Department of Medicine, Division of Gastroenterology, Duke University School of Medicine, Durham, NC

**Keywords:** colon ischemia, exercise, sildenafil, fludrocortisone, hematochezia

## Abstract

Colon ischemia is the most frequent form of intestinal ischemia. It is likely most commonly caused by mesenteric vasoconstriction in the microvasculature, though such vasoconstriction has never been documented, considering colonic blood flow has already normalized by the time of presentation. Long-distance running is a rare cause, presumably from blood shunting away from splanchnic vasculature. No effective treatment has previously been reported. We present a 41-year-old woman with recurrent abdominal pain and hematochezia after exercise. Initial workup was unrevealing, prompting a clinical diagnosis of exercise-induced colon ischemia from transient, reversible mesenteric vasoconstriction. Treatment with the vasodilator sildenafil and fludrocortisone for blood pressure support resulted in symptom resolution and her ability to return to exercise without adverse gastrointestinal symptoms.

## INTRODUCTION

While colon ischemia is a well-described and common clinical condition, exercise-induced colon ischemia (EICI) is a rare occurrence that has only sporadically been reported in case literature.^1-4^ Furthermore, no effective treatment or prevention for EICI is known. We present a case of EICI and successful implementation of a novel therapeutic approach of sildenafil and fludrocortisone.

## CASE REPORT

A 41-year-old healthy woman was referred to gastroenterology clinic for evaluation of abdominal pain during long-distance running. She took no medications and was a longtime runner. Three months before her first gastroenterology clinic visit, she increased her regular run from 5 miles to 9 miles, which resulted in the new onset of cramping lower abdominal pain and hematochezia with clots, prompting her to scale back to the shorter distance, which was again well tolerated. Vitals signs were notable for a blood pressure of 135/78, pulse rate of 68, normal temperature, and body mass index of 23.67 kg/m^2^. Physical examination after a longer run revealed mild epigastric and periumbilical tenderness. She denied family or personal history of hypercoagulability or inflammatory bowel disease. Laboratory results were notable for a normal complete blood count with a hemoglobin of 12.3 g/dL, a normal complete metabolic panel, and normal thyroid studies. Lactate was not sent given the patient did not present during acute episodes. Computed tomography (CT) angiography of the abdomen and pelvis including a mesenteric ischemia protocol was normal. A presumed diagnosis of EICI was made. She was advised to limit the distance of her runs and to ensure proper hydration before activities. However, her symptoms continued for several months, with attempts at shorter distances now resulting in episodes of abdominal pain and hematochezia. Eventually, she had to avoid all exercise, causing significant decline in her quality of life and mental health.

The patient returned to gastroenterology clinic for further evaluation. Upper endoscopy and colonoscopy were performed and were unremarkable, including histologic examination of random gastric, duodenal, terminal ileal, and colonic biopsies. Rheumatologic consultation was obtained; concern for vasculitis was low. At her next gastroenterology clinic visit 9 months later, the patient also mentioned weakness and occasional lightheadedness before exercise, prompting cardiology consultation for evaluation of hypotension-related colonic hypoperfusion. Cardiology recommended drinking 6 to 8 cups of water daily and supplementing electrolytes with an over-the-counter sport drink containing 400 mg of sodium (17% daily value) for improved hydration and blood pressure support. Stress echocardiography was also obtained and unremarkable.

Early the following year, with ongoing symptoms and inability to perform meaningful exercise, the patient began a therapeutic trial of sildenafil. She started daily sildenafil 25 mg 20 minutes before running. However, after 3 days of this medication, she experienced intolerable lightheadedness, leading to sildenafil cessation. Over the next 2 years, she had to limit herself to light exercise for a maximum of 30 minutes daily to avoid symptoms. She did not present for medical evaluation during this interval, limiting herself to light exercise while still enduring intermittent episodes of abdominal pain and hematochezia. However, following an episode of severe abdominal cramping and hematochezia after only a short walk, the patient returned to gastroenterology clinic. Hemoglobin level remained normal. It was decided to retrial sildenafil at a lower dose, 12.5 mg, before exercise together with intensive hydration. Fludrocortisone 0.05 mg daily before exercise was added to augment blood pressure and was tolerated without hypertension, hypokalemia, or edema.

**Figure 1.**
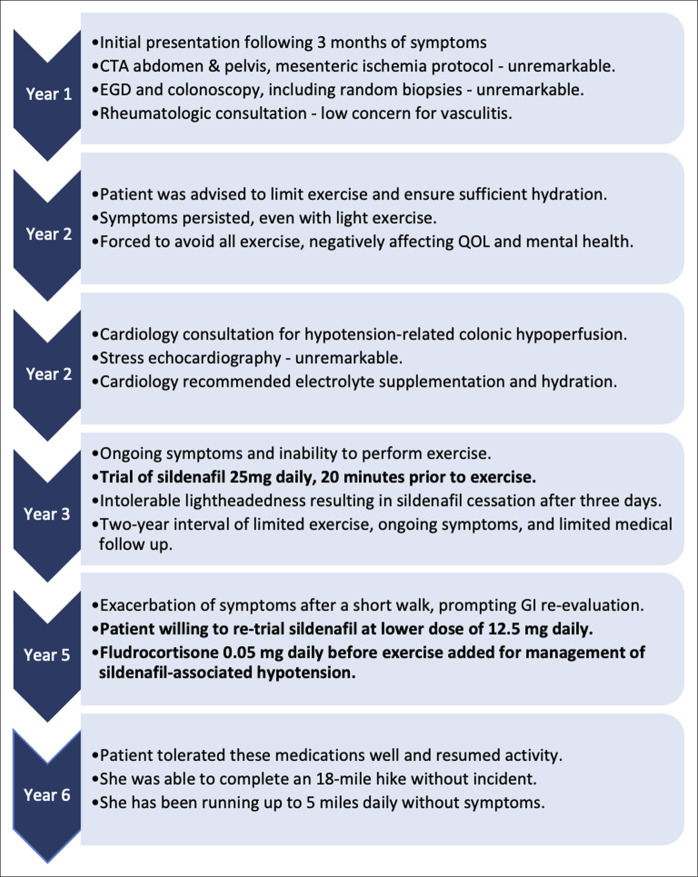
Summary of case events. CTA, computed tomography angiography; EGD, esophagogastroduodenoscopy; GI, gastrointestinal; QOL, quality of life.

She tolerated these medications well, and 1 month later, she was able to resume running without symptoms. The patient completed an 18-mile hike without incident and returned to regular running, though limiting herself to 5 miles, with longer distances or hot weather triggering mild abdominal cramping. Two years later, she has successfully continued on this medication regimen. She is able to engage in moderate physical activity with sustained symptom control. A summary of case events is provided in Figure 1.

## DISCUSSION

Colon ischemia, the most common form of intestinal ischemia, can be precipitated by vigorous exercise.^5,6^ Prevalence of gastrointestinal (GI) symptoms among athletes has varied in the published literature. One cohort study on runners of varying abilities found that 3% reported serious GI symptoms, such as nausea, vomiting, or diarrhea, with just 4 (0.3%) athletes reporting bloody stools either during the run or the day after.^7^ Another study on runners noted diarrhea in 26% and fecal urgency in 54%, while a third study involving endurance athletes found that 61% of participants reported lower GI symptoms.^8,9^ Among a cohort of marathon runners, 6% reported overt hematochezia.^10^

GI symptoms were associated with female sex, dehydration, and inability to complete the planned run. Another study identified a 6% prevalence of hematochezia among marathon runners. Patients with suspected EICI should be evaluated for hypercoagulability, medication effects, and risk factors of hypovolemia, such as high ambient temperatures or limited hydration. Angiography or CT angiography should be considered to rule out possible mesenteric stenosis if colon ischemia is noted on colonoscopy or CT scan especially when only the right side of the colon is involved. Other causes of rectal bleeding, such as infectious colitis, polyp or colorectal cancer, and inflammatory bowel disease should also be evaluated with laboratory tests and/or colonoscopy if such episodes occur at times other than just during exercise. The occurrence of abdominal pain and bloody bowel movements or hematochezia within a few hours after a long run strongly suggests the diagnosis of EICI.^4^ Wall thickening of the cecum or proximal colon was the most common CT finding in a series of 3 marathon runners presenting acutely with reversible colon ischemia; descending and sigmoid colon were involved in one of the 3 cases.^11^ While colonoscopy is not typically performed in acutely ill patients with colon ischemia, endoscopists may encounter thumbprinting (subepithelial hemorrhage and edema), ulceration, or other nonspecific signs of mucosal injury.^12^ Colonoscopy in active long-distance runners who presented within a few days after developing exercise-induced abdominal pain and hematochezia described findings of patchy mucosal bleeding, edema, erosion, and friability, as one would expect, regardless of the cause of the ischemic injury.^3,6,13-15^

A number of studies have attempted to evaluate the splanchnic vasculature as well as intestinal and colonic function during vigorous exercise, and suggest that EICI may be caused by physiologic shunting blood from the mesenteric bed to the muscles triggered by increased sympathetic tone during intensive exercise, especially with associated dehydration.^3,16-18^ The result is reversible, transient hypoperfusion of the mesenteric blood supply with subsequent colonic injury.

Existing case literature suggests treatment using intravenous hydration and brief observation, with more severe cases requiring admission for medical intensive care or even surgical management with colectomy.^1,2^ Apart from emphasizing the importance of appropriate pre-exercise hydration, existing literature does not offer additional preventive strategies. For patients experiencing persistent EICI despite appropriate hydration, further treatment strategies are warranted.

Based on the presumptive mechanism of splanchnic hypoperfusion, we speculated that sildenafil, a cGMP-specific phosphodiesterase (PDE-5) inhibitor that causes vasodilation, might result in improved mesenteric blood flow and prevention of exercise-associated colon ischemia. However, our patient developed symptoms of hypotension and therefore could not tolerate initial treatment with 25 mg sildenafil. Fludrocortisone, a corticosteroid with potent mineralocorticoid receptor activity, augments blood pressure by increasing sodium ion and water retention in the distal nephron. It was hypothesized that fludrocortisone could increase sildenafil tolerability. A trial of low-dose sildenafil was preferred over trialing alternate vasodilators at full-dose given similar risk of hypotension. Use of sildenafil 12.5 mg, 30 minutes before exercise, together with intensive hydration, electrolyte supplementation, and fludrocortisone 0.05 mg was better tolerated and successfully prevented symptoms. While aggressive hydration and electrolyte supplementation represent first-line adjunctive measures to address hypotension, we demonstrate the potential for fludrocortisone as a second-line option to increase blood pressure and reduce the side effect of lightheadedness. Notably, therapeutic benefit of sildenafil alone could not be assessed due to patient intolerance and rather could only be assessed in combination with fludrocortisone, raising the possibility of fludrocortisone alone providing therapeutic benefit. However, this is less likely given sildenafil's known mechanism of action.

It is important to note the off-label use of sildenafil and fludrocortisone in this case, as well as the potential side-effects of hypo- or hypertension, electrolyte abnormalities, edema, and other reported rare long-term effects. Addressing such side effects is likely key to ensuring tolerance of sildenafil and sustained patient uptake. Finally, while this patient responded to treatment with sildenafil and fludrocortisone, further cohort and trial data are needed before this treatment strategy can be more confidently generalized to a broader population. In the meantime, clinicians and patients considering trialing this treatment should proceed cautiously.

In conclusion, medical management with sildenafil, fludrocortisone, and hydration offers a new therapeutic option for prevention of colon ischemia in individuals planning vigorous exercise, such as long-distance running.

## DISCLOSURES

Author contributions: S.R.V. Gadi: manuscript preparation and edits. L.J. Brandt: critically revised manuscript. J. Roper: conceptualization and critically revised manuscript. J. Roper is the article guarantor.

Financial disclosure: None to report.

Previous presentation: Poster presentation at ACG 2024; October 28, 2024; Philadelphia, USA.

Informed consent was obtained for this case report.

## ABBREVIATIONS:

CT: computed tomography
EICI: exercise-induced colon ischemia
GI: gastrointestinal
PDE-5: phosphodiesterase 5
